# Born Too Soon: Learning from the past to accelerate action in the next decade

**DOI:** 10.1186/s12978-025-02044-8

**Published:** 2025-06-23

**Authors:** Anna Gruending, Joy E. Lawn, Amy Reid, Etienne V. Langlois, Bo Jacobsson, Lori McDougall, Rajat Khosla, Zulfiqar A. Bhutta, Ashok K. Deorari, Lily Kak, Judith Robb-McCord, Nabila Zaka, Ditas Duque Decena, Helga Fogstad, Anshu Banerjee, Mary V. Kinney, Queen Dube

**Affiliations:** 1 Partnership for Maternal, Newborn and Child Health (PMNCH), World Health Organization (WHO), Geneva, Switzerland; 2 Maternal, Adolescent, Reproductive & Child Health (MARCH) Centre, London School of Hygiene & Tropical Medicine, London, UK; 3 Department of Obstetrics and Gynecology, University of Gothenburg, Gothenburg, Sweden; 4 Centre for Global Child Health, The Hospital for Sick Children, Toronto, Canada; 5 Himalayan Institute of Medical Sciences, Swami Rama Himalayan University, Dehradun, India; 6 USAID, Washington, USA; 7 Sabin Vaccine Institute, Washington, USA; 8 UNICEF, Islamabad, Pakistan; 9 Department of Obstetrics and Gynecology, University of Santo Tomas Hospital, Manila, Philippines; 10 UNICEF, New York, USA; 11 Department of Maternal, Newborn, Child and Adolescent Health and Ageing, World Health Organization, Geneva, Switzerland; 12 University of the Western Cape, Cape Town, South Africa

**Keywords:** Newborn, Neonatal, Preterm birth, Policy, Innovation, Evidence, Social mobilisation, Community engagement, Polycrisis, Climate change, Conflict, Pandemic, Cost-of-living, Continuum of care

## Abstract

**Progress:**

This paper is a narrative review that takes stock of the progress in addressing preterm birth over the past decade – notably on policies, national plans, innovation, evidence, social mobilisation, and community engagement – to inform future progress on preterm birth.

At the global policy level, many countries have strongly supported collective initiatives and resolutions on maternal and newborn health relevant to preterm birth in multilateral fora, most recently through a World Health Assembly resolution calling for a revival amongst the global community on stalled progress for maternal, newborn and child health. Following the adoption of other global plans, like the Every Newborn Action Plan and Strategies for Ending Preventable Maternal Mortality, most countries set corresponding national mortality and coverage targets, and many have national and subnational policies and plans for integrated maternal and newborn health. Adequate financing remains a challenge, and sexual and reproductive health and rights of women and girls are being challenged globally.

There have been significant advances in evidence-based interventions for preterm birth prevention and care, reflected in updated World Health Organization guidelines on antenatal, intrapartum and postpartum care, and care for small and sick newborns. The past decade has also seen progress in social mobilisation and community engagement, particularly parent groups and healthcare professional organisations advocating on issues surrounding preterm birth.

**Polycrisis and vulnerability:**

There are, however, significant challenges that continue to hamper progress on preterm birth. Polycrisis – the interplay of overlapping economic, geopolitical, and environmental crises – compounds existing inequities, especially in places where health systems are already weak. Distinct and overlapping threats from conflict, climate change and the cost-of-living crisis present life-or-death challenges to those already facing extreme vulnerability, particularly women and girls, and small and sick newborns.

**Preterm birth: a marker of maternal and neonatal health progress in the coming decade:**

The detrimental impacts of preterm birth are felt along the life course and across generations. The success of countries and the global community in preventing preterm births and ensuring high-quality care for mothers and preterm babies serves as a critical measure of progress – or failure – in advancing global efforts to improve maternal and newborn health.

## Plain language summary

Countries have agreed on global plans and resolutions to improve maternal and newborn health. The World Health Assembly recently emphasized the need for renewed efforts in this area. Many countries have set national goals, but implementation remains a challenge as does funding.

There have been advances in evidence-based interventions for preterm birth prevention and care, reflected in updated World Health Organization guidelines. Progress has also been made in community engagement, with parent groups and healthcare organisations as leading advocates. However, overlapping crises hinder progress, including conflicts, climate change, pandemics, and the cost-of-living crisis. These crises increase inequities and pose serious risks to women and newborns, especially where health systems are weak.

Preterm birth is an important indicator of progress on maternal and neonatal health. How well countries and the global community address preterm birth and provide care for mothers and babies will show their overall success or failure in improving maternal and newborn health more broadly.

## Key findings

• Over the past decade, global and national policy frameworks, targets and plans relevant to preterm birth have been adopted, though significant funding gaps remain.

•  Knowledge of ‘what works’ to prevent and delay preterm birth and to care for babies who are born preterm has improved and is reflected in updated World Health Organization technical guidelines.

•  The world has flatlined on preterm birth prevention resulting in renewed urgency to learn from and fully leverage knowledge gained over the past decade to accelerate progress in the next decade.

•  Overlapping and compounding challenges of climate change, conflict, pandemics and the cost-of-living crisis pose significant direct and indirect risks for pregnant women and preterm babies and must be mitigated.

•  Addressing preterm birth is a key component of integrated maternal and newborn health across the continuum of care and along the life course, and a key marker of progress.

## Background

More than a decade ago, a global coalition of diverse partners came together and launched *Born Too Soon: The Global Action Report on Preterm Birth* in 2012 [1]. The report shone a spotlight on preterm birth, gaining major media and policy attention all over the world, making the case for the needs of women and their newborns together, given the interconnectedness of their health outcomes, and the care needed for both.

Despite that big spike in attention, the burden of preterm birth has not shifted. This paper is the first in a series of seven papers adapted from the second *Born Too Soon* report, [2] published by PMNCH and partners in 2023 with major media coverage and attention [3]. In this paper, we provide a narrative review of progress since that first report in 2012, primarily from a policy lens, and consider challenges that have affected progress, including the polycrisis, with preterm babies being the most vulnerable. We then look to the future, considering the issue of preterm birth as a public health lynchpin for more rapid and integrated progress for women and children’s health more broadly, and outline some conceptual shifts that can help to accelerate progress.

### Progress: looking back to inform our future

**Fig. 1:**
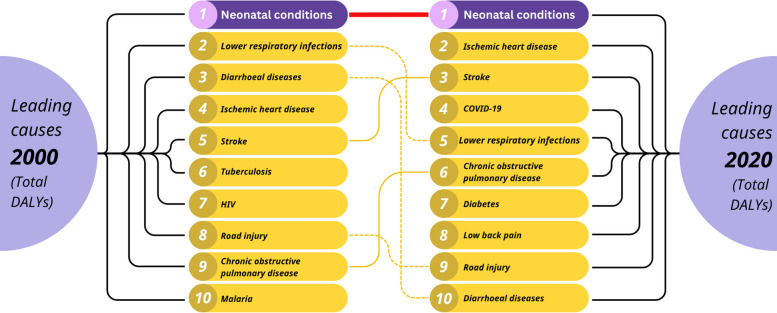
Neonatal disorders: the leading condition of global burden of disease, 2000 and 2020

Worldwide, 1 in 10 babies is born preterm (< 37 weeks’ gestation) – that’s an estimated one baby every two seconds. Rates of preterm birth have not changed during the past decade, and in some places, rates are rising [4]. Preterm birth has remained at the top of the Global Burden of Disease for 30 years – both in high- and low-income countries – making it a truly universal issue. Neonatal conditions are the leading cause of lost human capital in the most recent estimates of the Global Burden of Disease, measured in disability-adjusted life years (DALYs), and unchanged since 1990 (Fig. 1) [5]. In comparison, from 2000 to 2020, there have been improvements in other key drivers of child mortality and morbidity – namely lower respiratory infections and diarrheal diseases.

**Fig. 2:**
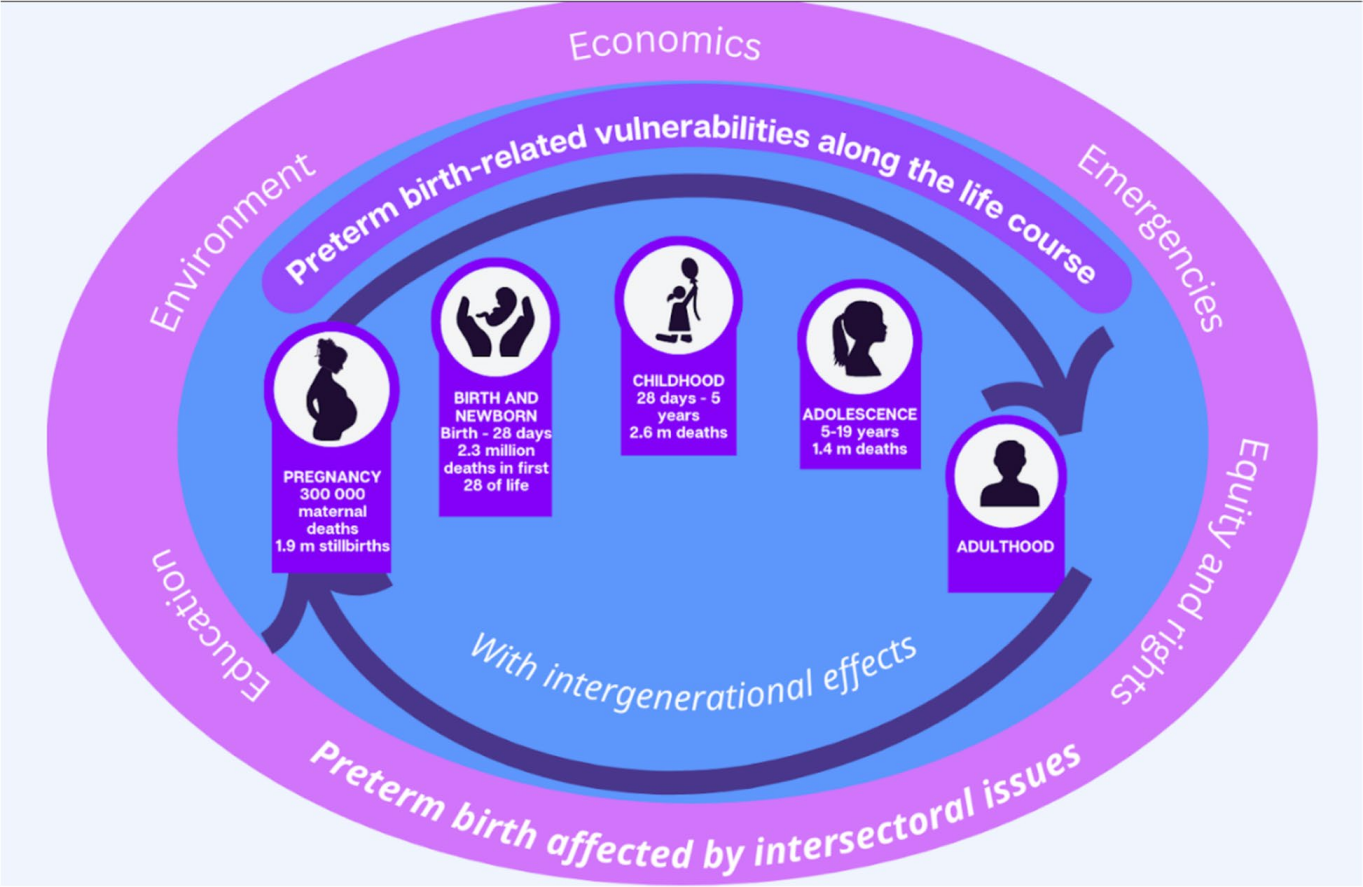
Preterm birth-related vulnerabilities along the life-course, across generations, and impacted by other sectors

When considering the life cycle, the risk for death and disability is greatest around the time of birth, and there are life-time risks associated with preterm birth and intergenerational effects (Fig. 2). In 2023, there were still more than 2.3 million neonatal deaths (17 per 1,000) [6] and 1.9 million stillbirths (14 per 1,000 births); [7] and in 2020, 287 000 maternal deaths [8]. In 2020 it is estimated that nearly 1 million newborns died due to complications of preterm birth (one baby every 40 s), and millions more survive with disabilities that follow them and their families throughout their lives. Preterm birth is the single largest killer of children under five years of age, accounting for more than one in three of all neonatal deaths (first month of life) [9]. Preterm birth can also contribute to an intergenerational cycle of ill-health, as babies born preterm have a higher likelihood of also giving birth to preterm babies [10, 11].

Additional adverse effects due to preterm birth are experienced across the life course. For a newborn, adverse effects include low birthweight, small for gestational age, poor perinatal outcomes, and increased mortality. Many newborns who survive preterm birth require intensive and specialised care, and some have disabilities that follow them throughout their lives – including increased risks of cerebral palsy, cognitive impairment, sight and hearing impairments and epilepsy [12]. Of 13 million preterm survivors annually, it is estimated that 345,000 have moderate to severe neurodevelopmental impairment, mostly in middle income countries, and an additional 567,000 have mild neurodevelopmental impairment [13]. For children and adolescents, adverse effects may also include physical health concerns, increased mortality, and social and behaviour challenges. For adults, the lingering effects of preterm birth can lead to chronic health conditions as well as higher risks of pregnancy complications [14]. In addition to showing the risks along the life course, Fig. 2 includes intersectoral issues outside of the health system that also influence preterm birth and small and sick newborn outcomes [15].

Beyond individuals, preterm birth has a deep and reverberating impact on families and communities. Parents and families of preterm babies experience high levels of stress and anxiety, [16, 17] and often devastating financial consequences following significant out-of-pocket expenses, including bankruptcy [18].

Today, the global community has the evidence, know-how, and technologies and systems to help preterm babies survive and thrive, yet an immense survival gap persists for babies born too soon, with 90% of extremely preterm babies (< 28 weeks) surviving in high-income countries and only 10% surviving in low-income countries [4, 19]. Worldwide, women, babies and their families are not uniformly getting the high-quality, respectful care that they need, and that is their right, to survive and thrive [18].

**Fig. 3:**
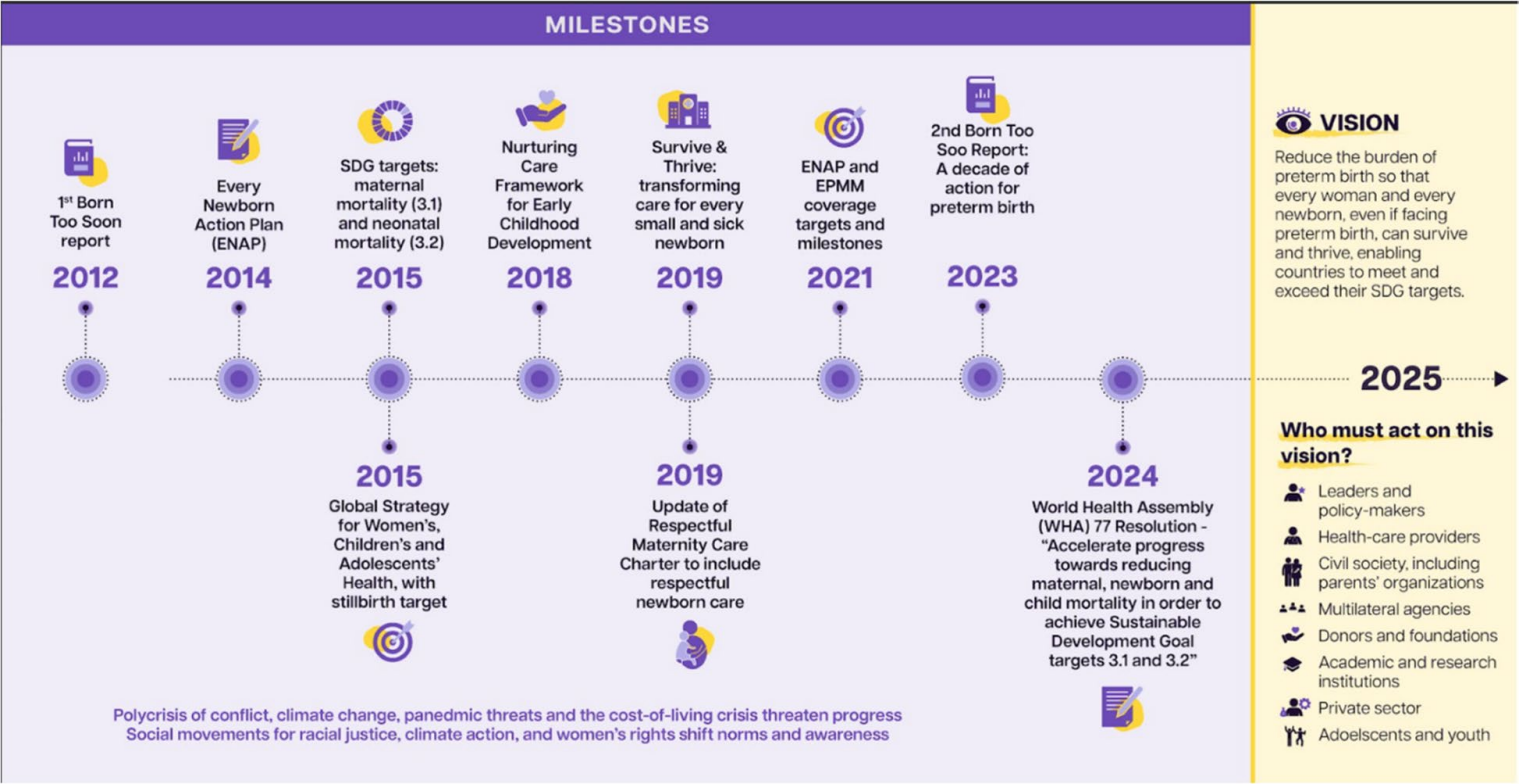
Timeline of progress from the past decade and vision for the next decade

Despite these significant challenges, progress has been made on certain fronts in the past decade. For example, between 2000–2019, improvements in eight maternal, neonatal, child and infectious conditions, including preterm birth complications, accounted for a 59% increase in global life expectancy, and in sub-Saharan Africa they accounted for 92% increase in life expectancy [20]. Reviewing this progress – notably on policies, national plans, innovation and evidence, and social mobilisation and community engagement – can help to inform future progress on preterm birth. Figure 3 summarizes some highlights of progress over the past decade.

### Policies

At the global policy level, there has been a groundswell of action over the past decade that has resulted in countries adopting numerous plans and resolutions relating to preterm birth. The Every Newborn Action Plan (ENAP), endorsed by the World Health Assembly in 2014, set the global target of 10 or fewer newborn deaths per 1,000 live births, and 10 or fewer stillbirths per 1,000 total births by 2035 and laid out clear actions for Member States to take to achieve these targets [21]. The Ending Preventable Maternal Mortality (EPMM) initiative followed in 2015, outlining maternal health targets for the Sustainable Development Goals (SDGs) and broad strategies for strengthening maternal health programmes using a human rights approach [22]. These two movements, which both have a significant focus on reducing inequities between and within countries, joined forces to advocate for action and to track progress, currently under the banner of ‘Every Woman, Every Newborn, Everywhere’ (EWENE) [23]. The SDGs, adopted in 2015, built upon the ENAP and EPMM targets and strategies to reduce newborn, under-five and maternal mortality. The Global Strategy for Women’s, Children’s and Adolescents’ Health, launched alongside the SDGs in 2015, further crystallised the actions required to meet these targets and included a stillbirth reduction target, which is absent from the SDG framework [24]. Additional policy frameworks, such as the Nurturing Care Framework for Early Childhood Development, adopted by the World Health Assembly in 2018, have recognized preterm birth as an important issue on which progress must be made to unlock other health benefits [25].

In 2024, countries articulated their commitment to these targets and goals through a resolution at the 77 th World Health Assembly (WHA) entitled: “Accelerate progress towards reducing maternal, newborn, and child mortality to achieve Sustainable Development Goals 3.1 and 3.2.” [26]. Led by the government of Somalia and co-sponsored by 51 Member States, the resolution called for a revival amongst the global community on stalled progress for maternal, newborn and child health. This resolution explicitly requests that the World Health Organization (WHO) develop relevant guidance on reducing preterm birth and stillbirths. To accompany this resolution, partners identified “six actions in six years” to prioritise including leadership, investments along the life-course, health care packages with the greatest impact, subnational action, data systems, and multisectoral partnerships [27].

The global political landscape impacting maternal and newborn health has been highly variable over the past decade. The election of socially conservative leaders, notably the United States of America in 2016 and again in 2024, has paved the way for the roll-back of basic sexual and reproductive health and rights. A coalition of countries have mobilised at a global level to coordinate their pushback to sexual and reproductive health and rights (SRHR) in multilateral fora, threatening gains on reproductive rights and gender equity [28].

However, this pushback also led to a renewed commitment and coordinated response. For example, “SheDecides” was launched by Ministers from the Netherlands, Belgium, Denmark and Sweden to mobilise politically and financially as in response to US President Donald Trump’s reinstatement and expansion of the Global Gag Rule – also known as the Mexico City Policy – in January 2017 [29]. In the past decade, a growing number of countries have embraced the banner of ‘feminist foreign policies’ – beginning with Sweden in 2014, and followed by Canada (2017), France (2019), Mexico (2020), Spain (2020) and others, many of which include support for SRHR as a cornerstone [30].

Some countries have also taken important domestic action to cement the protection of SRHR in their national policies and legislation. For instance, following an overwhelming majority in the National Assembly, in March 2024 France became the first country to explicitly include the right to abortion in its constitution [31].

### National plans

Following from global resolutions and targets on newborn and maternal health, most countries have set corresponding national mortality targets, and many have national and subnational policies and plans. In 2023, WHO, UNICEF and UNFPA published the results of a joint ENAP-EPMM tracking tool that found 83% of 106 countries had established national targets for reducing the maternal and newborns mortality rates. However, adequate financing remains a challenge. Only 61% of reporting countries had costed their maternal and newborn health plans, and only 12% reported that these plans were fully financed [32]. Donor funding for reproductive, maternal, newborn, child and adolescent health is estimated at approximately US$ 15.9 billion annually; however, funding for interventions explicitly targeting neonatal outcomes is less than 1% of this total allocated, and a mere 0.003% addressing terms associated with stillbirths [33]. As discussed elsewhere in this supplement, political will and sustainable financing remain major impediments to progress [34].

**Fig. 4:**
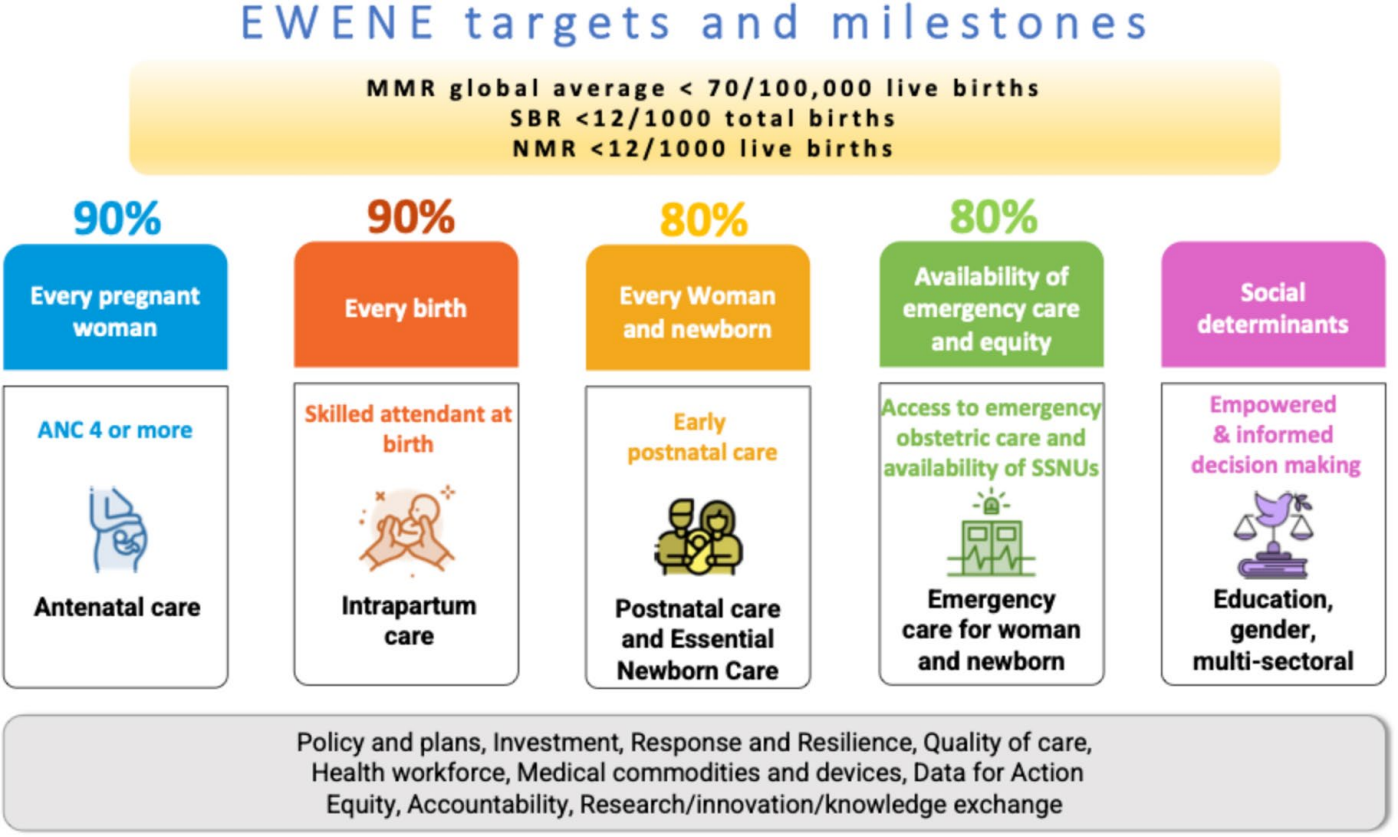
EWENE targets and milestones – 90/90/80/80

EWENE, together with country governments, have also established population coverage targets for five high impact packages of care, known as 90–90-80–80: 90% coverage of four or more antenatal care contacts (ANC4); 90% of births attended by skilled birth attendants (SBA); 80% early routine postnatal care; and 80% of districts with at least 70% ANC4, 80% SBA, and 60% PNC coverage (Fig. 4) [32]. The sub-national targets are particularly important given that wide disparities within countries are often masked by national averages. The global MNH community has united around these targets, but rates of improvement must accelerate if the coverage targets are to be achieved by 2025. Further, ensuring respectful and quality care for pregnant women, new mothers and newborns remains a critical gap [32].

### Innovation and evidence

The past decade has seen significant advancements in the evidence base for preterm birth prevention and care, and the reduction of stillbirths. These advances – including, for example, new evidence on the use of antenatal corticosteroids, on kangaroo mother care and on the implementation of small and sick newborn care (SSNC) – are reflected in updated WHO guidelines on antenatal, [35] intrapartum, [36] and postpartum [37] care, and care for small and sick newborns, [38] including for preterm and low birthweight babies [39] and are reviewed in detail in other papers in this supplement [19, 40]. These guidelines also bring forward evidence to support the importance of women’s agency and preferences, as well as people-centred care for more positive childbirth experiences and outcomes for newborns and their families. Notably, keeping the mother and baby together and family involvement in routine care for preterm and low-birthweight babies is recommended, having been shown to improve health outcomes, increase breastfeeding practices, decrease parental anxiety, and shorten hospital stays [39]. Evidence on the impact of community health workers in addressing neonatal mortality and morbidity has also expanded, notably demonstrating their potential to improve health equity and child and maternal health outcomes among vulnerable populations, including ethnic minorities, immigrants and rural communities [41]. This is part of a growing evidence base that recognizes the importance of community engagement and social participation for positive maternal and newborn health outcomes.

There are also new opportunities arising through innovation, including new technologies, such as artificial intelligence (AI), to strengthen data systems and accountability for maternal and newborn health, including newborn screening for birth defects, along with digital interventions that have demonstrated impact in improving health outcomes in the short and long term [42]. However, these technological advancements also raise challenges for users and policymakers. The intersection of SRHR and AI presents challenges on data governance, transparency, equity, and accountability, but also open up possibilities to accelerate people-centred care and strengthen the quality of care, in addition to facilitating the uptake of health information, promotion and education, advancing screening and diagnosis processes, and improving health systems management [43].

### Social mobilisation and community engagement

The past decade has also seen progress in social mobilisation and community engagement, particularly parent groups and healthcare professional organisations advocating on issues surrounding preterm birth [18]. Parent groups have multiplied, [44] particularly in the global north but also in the global south, and have built networks to advocate for their rights, including parental involvement in decision-making and provision of care for preterm babies [45]. The global awareness day, World Prematurity Day on November 17 th each year, has grown in size for nearly two decades with involvement from more than 200 organisations in over 100 countries joining forces with activities and special events in 2023 [46]. A global parent/patient group, the Global Alliance for Newborn Care (GLANCE), was established in 2018. Despite this progress, a robust civil society movement for maternal and newborn health, including for preterm birth, remains a gap in the global health architecture.

We have also witnessed other considerable shifts, many broad and societal, that have profoundly altered the landscape for maternal and newborn health. Grassroots activism has snowballed into widespread global movements for the defence of women’s rights and bodily autonomy, for racial justice, and for climate action, to name a few. These movements have shifted norms of what is and isn’t acceptable and increased societal demands for both progress and accountability [18, 47].

For instance, the #BlackLivesMatter movement has increased public focus on structural racism in the United States of America and elsewhere that leads to striking racial disparities in maternal and newborn health within the African American population [48, 49]. According to the Centers for Disease Control, Black women in the US are 2.6 times more likely to die from pregnancy-related causes compared to White women, [50] and rates of preterm birth are 50% higher [51]. In recent years, this issue has been accorded higher political priority. In 2021, the Black Maternal Health Caucus in the USA introduced the ‘Momnibus Act,’ a multi-pronged initiative of 13 individual bills to tackle the maternal health crisis [52]. The impact of social movements on racial justice, reproductive rights and climate actions are further explored in other papers in this supplement, notably paper 3 [15, 18, 40].

## Polycrisis and heightened vulnerability for women and newborns

Despite progress on policies, plans, evidence and social mobilisation, the latest data on preterm birth reveals that progress has not gone fast enough or far enough, with rates of preterm birth remain largely unchanged between 2010 and 2020 [4]. Maternal and newborn deaths – the vast majority of which are preventable with high-quality care – are increasingly concentrated in a subset of fragile countries that experience often-overlapping crises [53, 54].

The term ‘polycrisis’ was coined two decades ago by philosopher and sociologist Edgar Morin, [55] and can be defined as a cluster of related global risks – economic, geopolitical and environmental [56] – with “compounding effects, such that the overall impact exceeds the sum of each part.” [57]. Even when distinct, in a globalised world, there are strong interdependencies between crises [58].

**Fig. 5:**
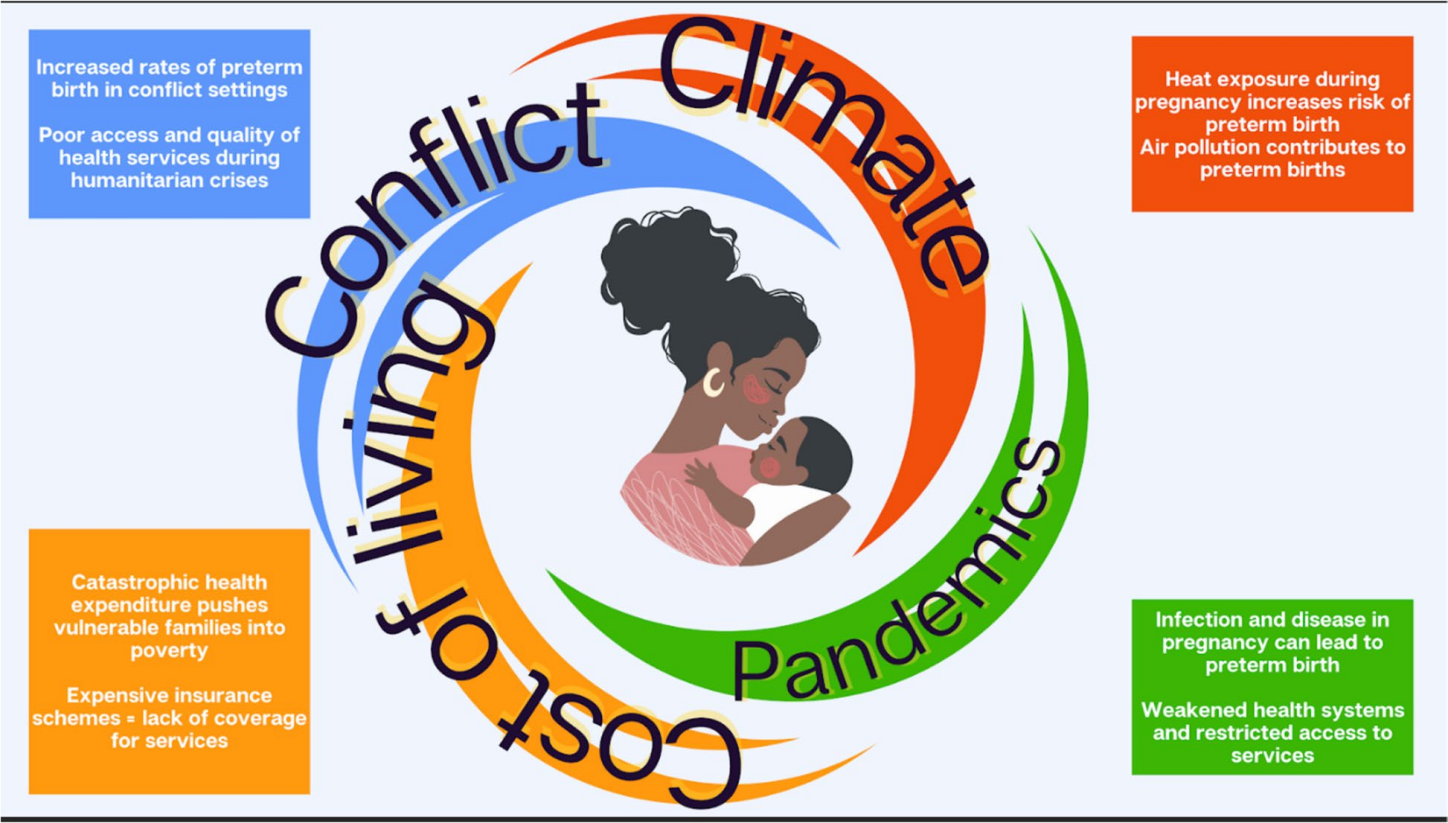
Impacts of polycrisis on preterm birth

Distinct and overlapping threats from conflict, climate change and the cost-of-living crisis compound existing inequities for those already facing extreme vulnerability, including women and girls, and small and sick newborns. Through various mechanisms both direct and indirect, polycrisis can increase the risk of preterm birth and limit the availability and quality of care when it does take place (Fig. 5).

### Climate change

Climate change has been identified as the greatest threat of the coming decade and beyond, [57] and its impacts on human health are long-term and wide-ranging [59]. Climate change impacts maternal and newborn health through both direct and indirect mechanisms. For instance, exposure to heat during pregnancy is associated with a higher risk of preterm birth, low birthweight, and stillbirth [60–63]. Air pollutants, such as methane and black carbon, contribute to both climate change and ill health [64]. It is estimated that, in 2019, air pollution contributed to 6 million preterm births and almost 3 million low-birth-weight babies [65]. A 2020 global report estimated that air pollution accounts for 20% of newborn deaths worldwide, mostly as a result of preterm birth and low birth weight [66].

Climate change has contributed to the recent surge in natural disasters, including record-breaking fires and unprecedented flooding. These extreme weather events can damage critical infrastructure and hinder access to essential maternal and newborn health services. Low-resource communities lack adaptive capacity and may be hardest hit [59]. Other indirect impacts include food insecurity and malnutrition due to drought, increased conflict and displacement driven by competition between communities for scarcening resources, and increases in vector-borne diseases due to their widening geographic spread [67].

Much of the data linking climate change to adverse maternal and newborn health outcomes is relatively recent, and merits urgent attention to mitigate the impact of these environmental risk factors on women and newborns. Paper 6 in this supplement further explores this emerging evidence base and makes recommendations for action [15].

### Conflict

By the end of 2023, more than 117 million people worldwide had been driven from their homes by war, violence, persecution and human rights abuses, [68] with women and children disproportionately affected [54]. In addition to deaths directly caused by conflict, the indirect impacts (e.g. collapsing health systems, restricted access) are often even greater, as health services may be disrupted by targeted damage to buildings, equipment, and infrastructure [54, 69–71]. Women trapped in conflicts are more at risk of exposures (nutritional deficiencies, untreated medical conditions, etc.) that contribute to preterm birth, low birth weight babies, and congenital conditions. Stress, insecurity, and malnutrition are also linked to higher incidence of preterm birth, in addition to long-term childhood development with potential consequences for future generations. Babies born too soon, too small, or with other health complications are especially vulnerable in conflict situations, especially those requiring secondary and tertiary care.

Neonatal intensive care units, reliant on continuous power, essential supplies, and trained staff, are particularly at risk [72]. Strikingly, 64% of global maternal deaths, 50% of newborn deaths, and 51% of stillbirths occurred in the 29 countries with UN Humanitarian Appeal in 2023 [73].

Paper 6 in this supplement expands on the complexity of preterm birth in conflict settings and other emergencies, while providing a synthesis of evidence-based interventions to uphold essential maternal and newborn health services and commodities in these settings [15].

### Pandemic threats

As noted by the Global Pandemic Monitoring Board, “our globalised world is prone to pandemics,” [74] though it took the COVID-19 pandemic to drive awareness of these risks to the top of the global agenda. Since 2023, the world has experienced outbreaks of Mpox, a resurgence of Ebola in Uganda, Nipah virus in South Asia, outbreaks of avian influenza, and new cholera outbreaks in several countries experiencing conflict and natural disasters [75]. There is an estimated 23% chance that another pandemic, with mortality on the scale of COVID-19, will occur in the next 10 years [20]. While epidemiological risk varies by disease, the broader tendency of pandemics to weaken already fragile health systems can wreak particular havoc for the most vulnerable members of society [76].

Pandemics pose both direct and indirect risks. For instance, contracting Mpox during pregnancy can pose direct risks for both mother and baby and can lead to miscarriage, stillbirth, and newborn death. Children may be at greater risk of severe Mpox than adults with corresponding higher mortality, and existing vulnerabilities (such as malnutrition) raise the risk of further complications [77].

The impacts of pandemics are also indirect. For example, the COVID-19 pandemic destabilised health services for women and newborns, and in many countries, led to a “separation crisis” with preterm babies routinely separated from their families. This harmful practice jeopardised high-impact practices such as kangaroo mother care (KMC) and exclusive breastfeeding that have been shown to significantly reduce neonatal mortality [78]. A study in 2021 estimated that if universal coverage of KMC were achieved, more than 125 000 newborn lives would be saved, whereas the risk of newborns catching and dying from COVID-19 would result in fewer than 2000 deaths [79]. However, despite this evidence, in many settings the damaging restrictions introduced or accentuated by the pandemic, including separation, remain in place.

### Cost-of-living crisis

The cost-of-living has been dramatically pushed up by disruptions to supply chains, caused by COVID-19, conflict and the climate crisis [80]. Global inflation rose from 4.7% in 2021 to 8.7% in 2022, with double-digit inflation in nearly half the world. While 2022 seems to have been the peak (the rate reduced to 5.9% in 2024), declines in inflation rates since 2022 have been much slower in low- and middle-income countries than in high-income countries, a trend that is projected to continue [81].

This crisis deepens the vulnerability of preterm babies and their families. There are reports of discharged babies returning to intensive care because families cannot afford heating and oxygen at home and, in a 2022 survey conducted in the UK, 84% of parents with a baby in neonatal care said the rising cost of living had curtailed their ability to travel to and from hospital [82]. The impact of this crisis on maternal and newborn health is yet to be fully measured and understood.

The vulnerability of women and newborns amidst the ongoing polycrisis risks further contributing to an intergenerational cycle of risk for preterm birth [10, 11].

## Looking ahead: preterm birth as a marker of progress in the coming decade

The latest data on preterm birth and maternal and newborn mortality are sobering, and the overarching global context does not inspire optimism. However, despite challenges, learning from the past decade demonstrates that change is possible and concerted advocacy initiatives, sometimes catalysed by influential reports like *Born Too Soon* in 2012, can influence progress [83]. Indeed, how countries and the global community fare on efforts to prevent preterm birth and to provide high quality care for mothers and babies when it does take place, can serve as an important marker of progress – or of failure – on global efforts on maternal and newborn health more broadly. This section sets out some of the conceptual shifts that the authors of the 2023 *Born Too Soon* report, and of this corresponding supplement, have adopted to reflect new learnings from the past decade and to contribute to greater impact in the coming decade.

### Conceptual shifts: widening focus for increased impact

#### Vulnerable newborns and stillbirths

**Fig. 6:**
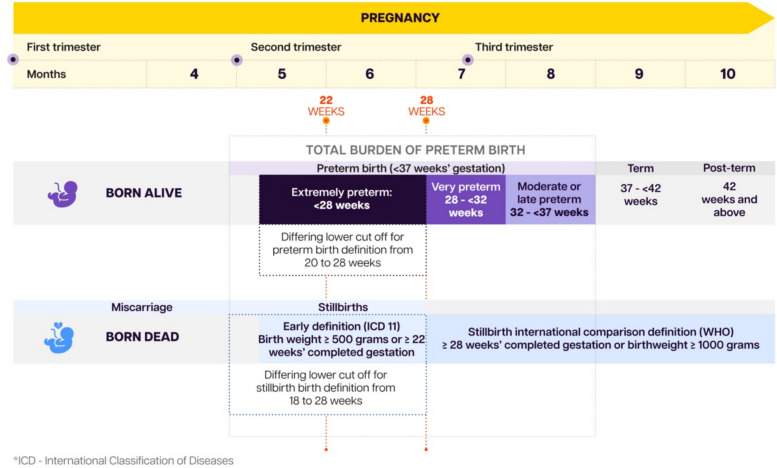
Definitions of preterm birth and related pregnancy outcomes

The first *Born Too Soon* report in 2012 focused on preconception care for women, care at birth, and care of preterm newborns (born before 37 completed weeks of gestation) [1]. The decade edition and this supplement focus on a larger group of vulnerable babies, including those with other neonatal complications, as well as stillbirths (Fig. 6). Small and sick newborns are treated in the same units by the same people, often require similar interventions.

The scope of this supplement also includes those born too small for gestational age, of whom there are a large number, particularly in South Asia. That said, the vulnerability of newborns is driven primarily by low gestational age (“born too soon”) causing the greatest risks of both death and disability. Babies who are both preterm and small for gestational age have the highest mortality risk but account for only about 1% of births worldwide [84]. The terminology used in this supplement is outlined in Table 1.

**Table 1:** Terminology used in the born too soon supplement

	**Definition**	**Terms used in this report**
Preterm birth	Liveborn baby at less than 37 completed weeks of gestation/pregnancy	“Born too soon”
Extremely preterm	Liveborn baby born at less than 28 completed weeks of gestations/pregnancy	
Small for Gestational Age	Live born baby that is under the 10 th centile for weight compared to expected for gestational age and sex	“Born too small”
Stillbirth	A baby born with no signs of life after 22 weeks of gestation according to International Classification of Diseases 11 definition	Late stillbirth (after 28 weeks of gestation, and used for WHO stillbirth estimates for international comparison) Early stillbirth before 28 weeks of gestation, and used for WHO stillbirth estimates for international comparison)
Vulnerable newborn	A liveborn baby that is preterm and/or small for gestational age	Small and sick newborn
Neonatal period	First 28 days after birth	Newborn
Neonatal intensive care	Intensive care of a newborn with complications	WHO level 3 newborn care, defined as including ventilation and more complex care
Small and sick newborn care	Care of newborns that are small (mainly preterm) and/or sick	WHO level 2 newborn care, defined as including basic respiratory support (Continuous Positive Airways Pressure or CPAP) and other special care including Kangaroo Mother Care

A baby born before 22 weeks of gestation with no signs of life is a miscarriage in ICD11

Source: Lancet Small and Vulnerable Newborn Series, 2023 [84, 85]

The supplement also explicitly includes stillbirths, which have too often been excluded from research and advocacy on preterm birth despite the clear links. Preterm labour can result in stillbirth, and intrauterine foetal death can result in preterm labour. New analyses suggest that around three-quarters of stillbirths after 22 weeks are preterm in high- and upper- middle income settings, yet these issues are rarely addressed together despite similar vulnerability pathways [84]. This issue is considered in more detail in paper 2 of this supplement. The.

### Continuum of care and beyond

**Fig. 7:**
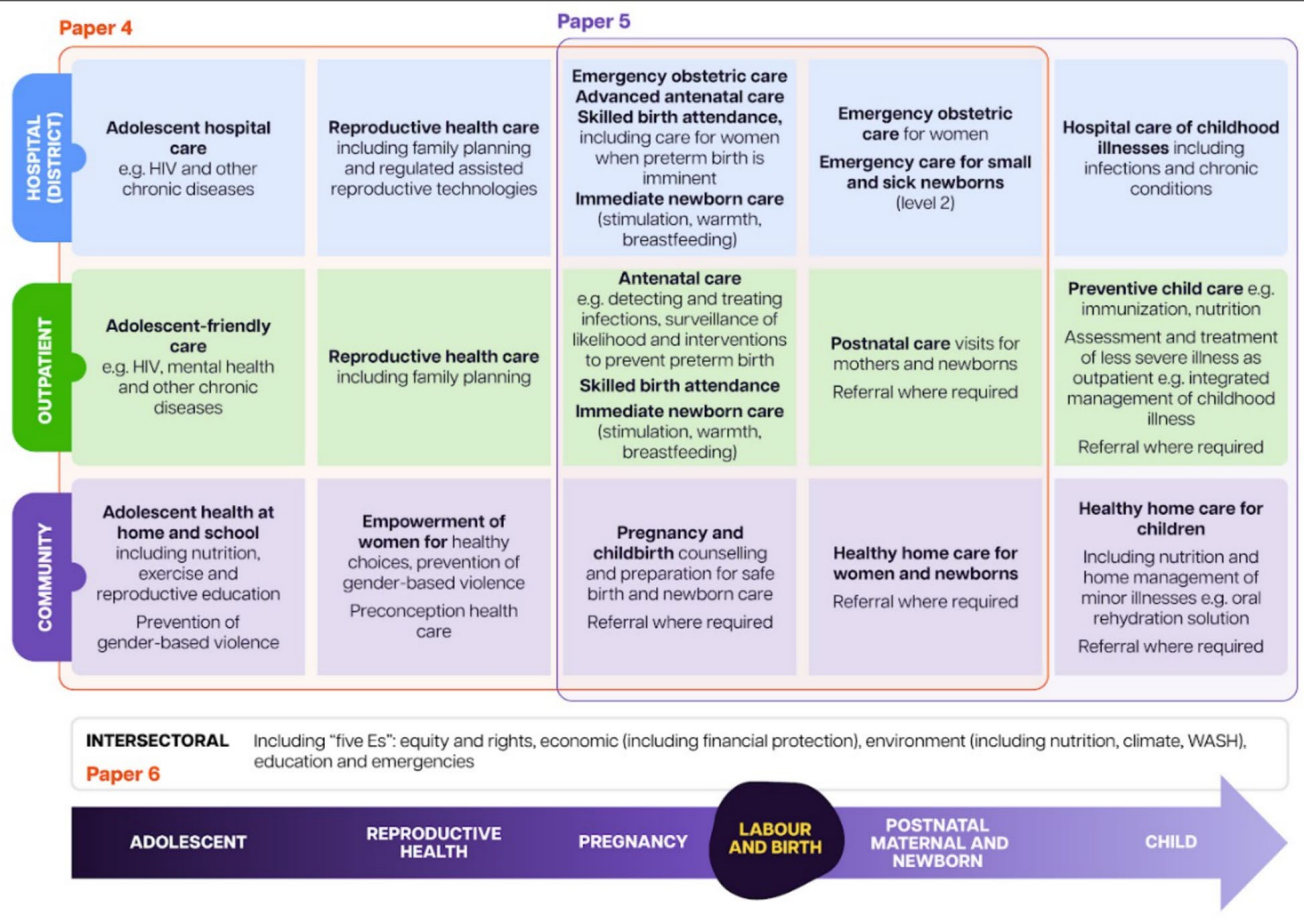
Addressing preterm birth along the continuum of care [86]

The first report was rooted along the continuum of care for mothers, newborns and children, and this supplement follows that same organising principle (Fig. 7) [86]. The continuum of care recognizes the life course approach, requiring a focus on SRHR, addressing the health needs of adolescent girls and women before, during and after pregnancy, and immediate and follow-up care for the newborn and child throughout their life cycle [86]. The continuum of care approach has proven cost-effective, including for the prevention and care of preterm birth [87–89]. In terms of the evidence base in this series, paper 4 focuses women’s health and maternal health services, [40] including pregnancy and birth; while, paper 5 focuses on care for small and sick newborns [19]. The papers in this supplement expand beyond the traditional continuum of care to include topics around rights and intersectoral action.

### Sexual and reproductive health and rights of adolescent girls

This supplement highlights the foundational importance of women’s sexual and reproductive health and rights, including maternal health [18, 40]. Compared to the 2012 *Born Too Soon* report, the 2023 edition and supplement place additional emphasis on adolescent girls, who have an increased risk of preterm birth but often have far less access to the services and care that they need to prevent unwanted pregnancies and to support their health and well-being. Unmet need for modern contraception is 43% among adolescent girls versus 24% for all women of reproductive age [90]. This has significant repercussions on the health and rights of adolescent girls as well as the health of their babies, who face a higher risk of preterm birth, low birth weight, and severe neonatal conditions [91]. Pregnancy and childbirth complications remain the second leading cause of death for 15–19 year old girls globally, [92] while adolescent pregnancies carry increased short and long-term health risks, including eclampsia and systemic infections [93]. As noted previously, adolescent pregnancy also contributes to an inter-generational cycle of risk for preterm birth.

### People-centred and rights-based approaches

The supplement also highlights the increased emphasis over the past decade on people-centred and rights-based approaches to preterm birth prevention and care [18]. At its most powerful, a human rights-based approach to preterm birth drives the development of policies and programmes that are respectful and of high quality, that involve and empower women and families, that consider the work environment and rights of healthcare providers, and that have firmly embedded accountability mechanisms. A shift in the past decade has also been the more explicit recognition of the mother-baby dyad, which includes the rights of women and newborns across all stages of the continuum of care [18].

### Care for survivors of preterm birth and their families

Finally, the supplement places additional emphasis on the follow-up care and support that is needed for survivors of preterm birth and their families over the course of their lives – a topic that has often been overlooked and under-appreciated [19].

## Conclusion

Evidence alone does not equal progress. However, robust evidence in the hands of committed actors is an important condition for momentum and action. The many partners that came together in 2023 to develop the decade edition of *Born Too Soon* hope that the revised report and this corresponding supplement, reflecting new data, evidence and policy initiatives, can rekindle commitment and propel action on preterm birth. Together, we must go faster and further to address the unacceptable burden of preterm birth.

## Data Availability

All data are available in the paper or in supplementary files. Additional information is available at www.borntoosoonaction.org.

## Abbreviations

AI: Artificial Intelligence
ANC: Antenatal care
DALY: Disability-adjusted life years
EFCNI: European Foundation for the Care of Newborn Infants
ENAP: Every Newborn Action Plan
EPMM: Ending Preventable Maternal Mortality
EWENE: Every Woman Every Newborn Everywhere
GLANCE: Global Alliance for Newborn Care
KMC: Kangaroo Mother Care
MNH: Maternal and newborn health
PNC: Postnatal care
SBA: Skilled birth attendance
SDG: Sustainable Development Goals
SRHR: Sexual and reproductive health and rights
WHA: World Health Assembly
WHO: World Health Organization

## References

[1] Born Too Soon. The Global Action Report on Preterm Birth. Geneva: World Health Organization; 2012.

[2] Born too soon. Decade of action on preterm birth. Geneva: World Health Organization; 2023.

[3] Langlois EV, Reid A, Lawn JE, Kinney MV, El Bizri M, Belizán JM, Gruending A, Jacobsson B. Born Too Soon: Priorities to improve the prevention and care of preterm birth. BMC Reprod Health. 2025;22(Suppl 2). 10.1186/s12978-025-02069-z PMC12186336 40555980

[4] Ohuma EO, Moller A-B, Bradley E, Chakwera S, Hussain-Alkhateeb L, Lewin A, et al. National, regional, and global estimates of preterm birth in 2020, with trends from 2010: a systematic analysis. The Lancet. 2023;402(10409):1261–71. 10.1016/S0140-6736(23)00878-4 37805217

[5] Global health estimates: Leading causes of DALYs. Geneva: World Health Organization; 2024 [Available from: https://www.who.int/data/gho/data/themes/mortality-and-global-health-estimates/global-health-estimates-leading-causes-of-dalys.

[6] Levels & Trends in Child Mortality. Report 2023, Estimates developed by the United Nations Inter-agency Group for Child Mortality Estimation. New York: United Nations Inter-agency Group for Child Mortality Estimation; 2024.

[7] Never Forgotten - The situation of stillbirth around the globe. New York: United Nations Inter-agency Group for Child Mortality Estimation 2022.

[8] Trends in maternal mortality to 2020: estimates by WHO, UNICEF, UNFPA. World Bank Group and UNDESA/Population Division. Geneva: World Health Organization; 2000. p. 2023.

[9] Liu L, Oza S, Hogan D, Chu Y, Perin J, Zhu J, et al. Global, regional, and national causes of under-5 mortality in 2000–15: an updated systematic analysis with implications for the Sustainable Development Goals. Lancet. 2016;388(10063):3027–35. 27839855 10.1016/S0140-6736(16)31593-8 PMC5161777

[10] Solé-Navais P, Flatley C, Steinthorsdottir V, Vaudel M, Juodakis J, Chen J, et al. Genetic effects on the timing of parturition and links to fetal birth weight. Nat Genet. 2023;55(4):559–67. 37012456 10.1038/s41588-023-01343-9 PMC10101852

[11] Svensson AC, Sandin S, Cnattingius S, Reilly M, Pawitan Y, Hultman CM, et al. Maternal Effects for Preterm Birth: A Genetic Epidemiologic Study of 630,000 Families. Am J Epidemiol. 2009;170(11):1365–72. 19854802 10.1093/aje/kwp328

[12] Taylor GL, O’Shea TM. Extreme prematurity: Risk and resiliency. Curr Probl Pediatr Adolesc Health Care. 2022;52(2):101132. 35181232 10.1016/j.cppeds.2022.101132 PMC9247808

[13] World Health Organization. Survive and thrive: transforming care for every small and sick newborn. 2019.

[14] Jacobsson B, Requejo JH, Dey T, Lavin T, Mannah MT, Menon R, Valencia C, Sharma G, Shennan A, Shakibazadeh E, Lumbiganon P, Bar-Zeev S, Steen S, Nguku A, Obara H, Noguchi L, Okong P, Singh SA, Sandall J, Gruending A, Qureshi Z, Vogel J. Born Too Soon: Women’s health and maternal care services, seizing missed opportunities to prevent and manage preterm birth. BMC Reprod Health. 2025;22(Suppl 2). 10.1186/s12978-025-02034-w PMC12186347 40556011

[15] Langlois EV, El Bizri M, Thompson K, Reid A, Khalil M, Gasparri G, Lawn JE, Dey T, Robb-McCord J, Benaskeur YI, Bonell A, Gidebo A, Scudder E, Kostelecky SM, Machawira P, Gronseth L, Prasad R, Sapkota D, SomaPillay P, Valsangkar B, Jacobsson B, Temmerman M. Born Too Soon: Integration of intersectoral interventions for impact on preterm birth. BMC Reprod Health. 2025;22(Suppl 2). 10.1186/s12978-025-02043-9 PMC12186354 40555977

[16] Pace CC, Spittle AJ, Molesworth CM, Lee KJ, Northam EA, Cheong JL, et al. Evolution of depression and anxiety symptoms in parents of very preterm infants during the newborn period. JAMA Pediatr. 2016;170(9):863–70. 27428766 10.1001/jamapediatrics.2016.0810

[17] Roque ATF, Lasiuk GC, Radünz V, Hegadoren K. Scoping Review of the Mental Health of Parents of Infants in the NICU. J Obstet Gynecol Neonatal Nurs. 2017;46(4):576–87. 28506679 10.1016/j.jogn.2017.02.005

[18] Kinney MV, Ateva E, Cocoman O, Schaaf M, Wanduru P, Khalil M, Sacks E, Tames R, Suguitani D, Stahlhofer M, Malhotra J, Ten Hoope Bender P. Born Toon Soon: Progress and priorities for respectful and rights-based preterm birth care. BMC Reprod Health. 2025;22(Suppl 2). 10.1186/s12978-025-02042-w PMC12186350 40555976

[19] Murless-Collins S, Ezeaka VC, Masoud NS, Walker K, Rhoda NR, Keenan W, Wall S, Bhutta ZA, Duran P, Bolaji O, Edmund K, Gupta G, Lawn JE. Born Too Soon: Care for small and sick newborns, evidence for investment and implementation. BMC Reprod Health. 2025;22(Suppl 2). 10.1186/s12978-025-02032-y PMC12188657 40555990

[20] Jamison DT, Summers LH, Chang AY, Karlsson O, Mao W, Norheim OF, et al. Global health 2050: the path to halving premature death by mid-century. The Lancet. 2024;404(10462):1561–614. 10.1016/S0140-6736(24)01439-9 PMC12320876 39419055

[21] SIXTY-SEVENTH WORLD HEALTH ASSEMBLY. WHA67.10 Newborn health action plan. Geneva: World Health Organization; 2014. Report No.: WHA67/2014/REC/1.

[22] Strategies toward ending preventable maternal mortality (EPMM). Geneva: World Health Organization; 2015.

[23] Every Women Every Newborn Everywhere: WHO, UNICEF, UNFPA; 2024 [Available from: https://ewene.org/.

[24] The Global Strategy for Women’s. Children’s and Adolescents’ Health (2016–2030). Geneva: World Health Organization; 2015. 10.2471/BLT.16.174714 PMC4850547 27147756

[25] Nurturing care for early childhood development. a framework for helping children survive and thrive to transform health and human potential. Geneva: World Health Organization, UNICEF, World Bank Group; 2018.

[26] Accelerate progress towards reducing maternal, newborn and child mortality in order to achieve Sustainable Development Goal targets 3.1 and 3.2. Geneva: World Health Organization; 2024. Report No.: A77/A/CONF./5.

[27] Six years to the SDG deadline. Six actions to reduce unacceptably high maternal, newborn and child deaths and stillbirths. Geneva: Partnership for Maternal, Newborn and Child Health; 2024.

[28] Conservative Member States Balk at References to ‘Gender’ in WHA Resolution [press release]. Geneva: Health Policy Watch 2024.

[29] The Story. SheDecides; [Available from: https://www.shedecides.com/our-story/.

[30] Feminist Foreign Policies. An Introduction. New York: UN Women; 2022.

[31] LOI constitutionnelle n° 2024-200 du 8 mars 2024 relative à la liberté de recourir à l'interruption volontaire de grossesse (1). Paris: Légifrance; 2024.

[32] Improving maternal and newborn health and survival and reducing stillbirth Progress Report. Geneva: World Health Organization; 2023.

[33] Kumar MB, Bath D, Binyaruka P, Novignon J, Lawn JE, Pitt C. Donor aid mentioning newborns and stillbirths, 2002–19: an analysis of levels, trends, and equity. Lancet Glob Health. 2023;11(11):e1785–93. 37858588 10.1016/S2214-109X(23)00378-9 PMC10603612

[34] Lawn JE, Khosla R, Reid A, Langlois EV, Kinney M, Gupta G, Mollel D, Jacobsson B, El Bizri M, Gruending A, Ruysen H, Thompson K, Ashorn P, McDougall L, Fogstad H, Shafique F, Banerjee A. Born Too Soon: accelerating change to 2030 and beyond. BMC Reprod Health. 2025;22(Suppl 2). 10.1186/s12978-025-02035-9 PMC12188660 40555992

[35] WHO recommendations on antenatal care for a positive pregnancy experience. Geneva: World Health Organization; 2016. 28079998

[36] WHO recommendations: intrapartum care for a positive childbirth experience. Geneva: World Health Organization; 2018. 30070803

[37] WHO recommendations on maternal and newborn care for a positive postnatal experience. Geneva: World Health Organization; 2022. 35467813

[38] Standards for improving the quality of care for small and sick newborns in health facilities. Geneva: World Health Organization; 2020.

[39] WHO recommendations for care of the preterm or low-birth-weight infant. Geneva: World Health Organization; 2022. 36449655

[40] Jacobsson B, Requejo JH, Dey T, Lavin T, Mannah MT, Menon R, Valencia C, Sharma G, Shennan A, Shakibazadeh E, Lumbiganon P, Bar-Zeev S, Steen S, Nguku A, Obara H, Noguchi L, Okong P, Singh SA, Sandall J, Gruending A, Qureshi Z, Vogel J. Born Too Soon: Women’s health and maternal care services, seizing missed opportunities to prevent and manage preterm birth. BMC Reprod Health. 2025;22(Suppl 2). 10.1186/s12978-025-02034-w PMC12186347 40556011

[41] WHO guideline on health policy and system support to optimize community health worker programmes. Geneva: World Health Organization; 2018. 30431747

[42] Recommendations on digital interventions for health system strengthening. Geneva: World Health Organization; 2019. 31162915

[43] The role of artificial intelligence in sexual and reproductive health and rights. Geneva: World Health Organization; 2024.

[44] Parent- and patient organisations. Global Alliance for Newborn Care; 2019 [Available from: https://www.glance-network.org/our-supporters/network/.

[45] Parent Organisations Summit. European Foundation for the Care of Newborn Infants; 2024 [Available from: https://www.efcni.org/parent-organisation-meeting/.

[46] World Prematurity Day. European Foundation for the Care of Newborn Infants; 2024 [Available from: https://www.efcni.org/activities/campaigns/wpd/.

[47] Choo EK, Byington CL, Johnson NL, Jagsi R. From #MeToo to #TimesUp in health care: can a culture of accountability end inequity and harassment? Lancet. 2019;393(10171):499–502. 30739670 10.1016/S0140-6736(19)30251-X

[48] Tobin-Tyler L. Black Mothers Matter: The Social, Political and Legal Determinants of Black Maternal Health across the Lifespan. SSRN Electronic Journal. 2021.

[49] Bailey ZD, Feldman JM, Bassett MT. How Structural Racism Works - Racist Policies as a Root Cause of U.S. Racial Health Inequities. N Engl J Med. 2021;384(8):768–73. 33326717 10.1056/NEJMms2025396 PMC11393777

[50] Maternal mortality rates in the United States, 2021, (2023).

[51] Black Maternal Health Week 2023: March of Dimes; 2023 [Available from: https://www.marchofdimes.org/black-maternal-health-week-2023.

[52] Lowe NK. Black Women’s Lives Matter. J Obstet Gynecol Neonatal Nurs. 2021;50(4):363–8. 34153227 10.1016/j.jogn.2021.06.001

[53] Protect the promise. 2022 progress report on the every woman every child global strategy for women’s, children’s and adolescents’ health (2016–2030). Geneva: World Health Organization; 2022.

[54] Bendavid E, Boerma T, Akseer N, Langer A, Malembaka EB, Okiro EA, et al. The effects of armed conflict on the health of women and children. The Lancet. 2021;397(10273):522–32. 10.1016/S0140-6736(21)00131-8 PMC7612212 33503456

[55] Morin E. Homeland Earth: A Manifesto for the New Millennium. London: Hampton Press; 1999.

[56] Lawrence M, Homer-Dixon T, Janzwood S, Rockstöm J, Renn O, Donges JF. Global polycrisis: the causal mechanisms of crisis entanglement. Global Sustainability. 2024;7:e6.

[57] The Global Risks Report 2023. Geneva: World Economic Forum; 2023.

[58] Prospects for Children in the Polycrisis: A 2023 Global Outlook. Internet. Florence: UNICEF; 2023.

[59] Roos N, Kovats S, Hajat S, Filippi V, Chersich M, Luchters S, et al. Maternal and newborn health risks of climate change: A call for awareness and global action. Acta Obstet Gynecol Scand. 2021;100(4):566–70. 33570773 10.1111/aogs.14124

[60] Chersich MF, Pham MD, Areal A, Haghighi MM, Manyuchi A, Swift CP, et al. Associations between high temperatures in pregnancy and risk of preterm birth, low birth weight, and stillbirths: systematic review and meta-analysis. BMJ. 2020;371:m3811. 33148618 10.1136/bmj.m3811 PMC7610201

[61] Bekkar B, Pacheco S, Basu R, DeNicola N. Association of Air Pollution and Heat Exposure With Preterm Birth, Low Birth Weight, and Stillbirth in the US: A Systematic Review. JAMA Netw Open. 2020;3(6):e208243. 32556259 10.1001/jamanetworkopen.2020.8243 PMC7303808

[62] McElroy S, Ilango S, Dimitrova A, Gershunov A, Benmarhnia T. Extreme heat, preterm birth, and stillbirth: A global analysis across 14 lower-middle income countries. Environ Int. 2022;158:106902. 34627013 10.1016/j.envint.2021.106902

[63] Lakhoo DP, Brink N, Radebe L, Craig MH, Pham MD, Haghighi MM, et al. A systematic review and meta-analysis of heat exposure impacts on maternal, fetal and neonatal health. Nature Medicine. 2024. 10.1038/s41591-024-03395-8 PMC11835737 39500369

[64] Hao H, Yoo SR, Strickland MJ, Darrow LA, D’Souza RR, Warren JL, et al. Effects of air pollution on adverse birth outcomes and pregnancy complications in the U.S. state of Kansas (2000–2015). Scientific Reports. 2023;13(1):21476. 38052850 10.1038/s41598-023-48329-5 PMC10697947

[65] Ghosh R, Causey K, Burkart K, Wozniak S, Cohen A, Brauer M. Ambient and household PM2.5 pollution and adverse perinatal outcomes: A meta-regression and analysis of attributable global burden for 204 countries and territories. PLoS Med. 2021;18(9):e1003718. 34582444 10.1371/journal.pmed.1003718 PMC8478226

[66] State of Global Air. Special Report. Boston: Health Effects Institute; 2020. p. 2020.

[67] Oberlin AM, Wylie BJ. Vector-borne disease, climate change and perinatal health. Semin Perinatol. 2023;47(8):151841. 37852894 10.1016/j.semperi.2023.151841

[68] Trends G. Forced displacement in 2023. Copenhagen: United Nations High Commissioner for Refugees; 2024.

[69] Wise PH. The Epidemiologic Challenge to the Conduct of Just War: Confronting Indirect Civilian Casualties of War. Daedalus. 2017;146(1):139–54.

[70] Keasley J, Blickwedel J, Quenby S. Adverse effects of exposure to armed conflict on pregnancy: a systematic review. BMJ Glob Health. 2017;2(4):e000377. 29333283 10.1136/bmjgh-2017-000377 PMC5706483

[71] Le K, Nguyen M. Armed conflict and birth weight. Econ Hum Biol. 2020;39:100921. 32846273 10.1016/j.ehb.2020.100921

[72] Lives of one million children ‘hanging by a thread,’ as child health services almost collapse across the Gaza Strip [press release]. UNICEF. 2023.

[73] Community-Based Maternal and Newborn Health Care Bringing care closer to home in humanitarian and fragile settings. New York: International Rescue Committee; 2023.

[74] Equity in pandemic preparedness. Geneva: Global Preparedness Monitoring Board; 2024.

[75] Fragile State A, of Preparedness,. Report on the State of the World’s Preparedness. Geneva: Global Preparedness Monitoring Board; 2023. p. 2023.

[76] Estimated Future Mortality from Pathogens of Epidemic and Pandemic Potential. London: Center for Global Development; 2023.

[77] Mpox. Geneva: World Health Organization; 2024 [Available from: https://www.who.int/news-room/questions-and-answers/item/mpox.

[78] Arya S, Naburi H, Kawaza K, Newton S, Anyabolu CH, Bergman N, et al. Immediate “Kangaroo Mother Care” and Survival of Infants with Low Birth Weight. N Engl J Med. 2021;384(21):2028–38. 34038632 10.1056/NEJMoa2026486 PMC8108485

[79] Minckas N, Medvedev MM, Adejuyigbe EA, Brotherton H, Chellani H, Estifanos AS, et al. Preterm care during the COVID-19 pandemic: A comparative risk analysis of neonatal deaths averted by kangaroo mother care versus mortality due to SARS-CoV-2 infection. EClinicalMedicine. 2021;33:100733. 33748724 10.1016/j.eclinm.2021.100733 PMC7955179

[80] The Lancet Public H. The cost of living: an avoidable public health crisis. The Lancet Public Health. 2022;7(6):e485. 35660204 10.1016/S2468-2667(22)00120-7 PMC9159733

[81] World Economic Outlook: Countering the Cost-of-Living Crisis. Washington, DC.: International Monetary Fund; 2022.

[82] Soaring cost of living leaves parents of sick children fearful about running medical equipment. London: Bliss for babies born premature or sick; 2022 [Available from: https://www.bliss.org.uk/news/2022/soaring-cost-of-living-leaves-parents-of-sick-children-fearful-about-running-medical-equipment#:~:text=Bliss%20has%20spoken%20to%20parents,early%20to%20afford%20rising%20costs.

[83] Darmstadt GL, Kinney MV, Chopra M, Cousens S, Kak L, Paul VK, et al. Who has been caring for the baby? Lancet. 2014;384(9938):174–88. 24853603 10.1016/S0140-6736(14)60458-X

[84] Lawn JE, Ohuma EO, Bradley E, Idueta LS, Hazel E, Okwaraji YB, et al. Small babies, big risks: global estimates of prevalence and mortality for vulnerable newborns to accelerate change and improve counting. Lancet. 2023;401(10389):1707–19. 37167989 10.1016/S0140-6736(23)00522-6

[85] Ashorn P, Ashorn U, Muthiani Y, Aboubaker S, Askari S, Bahl R, et al. Small vulnerable newborns-big potential for impact. Lancet. 2023;401(10389):1692–706. 37167991 10.1016/S0140-6736(23)00354-9

[86] Kerber KJ, de Graft-Johnson JE, Bhutta ZA, Okong P, Starrs A, Lawn JE. Continuum of care for maternal, newborn, and child health: from slogan to service delivery. Lancet. 2007;370(9595):1358–69. 17933651 10.1016/S0140-6736(07)61578-5

[87] Adam T, Amorim DG, Edwards SJ, Amaral J, Evans DB. Capacity constraints to the adoption of new interventions: consultation time and the Integrated Management of Childhood Illness in Brazil. Health Policy Plan. 2005;20(Suppl 1):i49–57. 16306069 10.1093/heapol/czi057

[88] Atrash HK, Johnson K, Adams M, Cordero JF, Howse J. Preconception care for improving perinatal outcomes: the time to act. Matern Child Health J. 2006;10(5 Suppl):S3-11. 16773452 10.1007/s10995-006-0100-4 PMC1592246

[89] Sepúlveda J, Bustreo F, Tapia R, Rivera J, Lozano R, Oláiz G, et al. Improvement of child survival in Mexico: the diagonal approach. The Lancet. 2006;368(9551):2017–27. 10.1016/S0140-6736(06)69569-X 17141709

[90] Adding It Up. Investing in Sexual and Reproductive Health 2019. New York: Guttmacher Institute; 2020.

[91] Adolescent pregnancy. Geneva: World Health Organization; 2024 [Available from: https://www.who.int/news-room/fact-sheets/detail/adolescent-pregnancy.

[92] The Global Health Observatory. Geneva: World Health Organization; [Available from: https://www.who.int/data/gho.

[93] Leftwich HK, Alves MV. Adolescent Pregnancy. Pediatr Clin North Am. 2017;64(2):381–8. 28292453 10.1016/j.pcl.2016.11.007

